# Medicare spending and use of subcutaneous biologic formulations with hyaluronidase

**DOI:** 10.1093/oncolo/oyaf149

**Published:** 2025-06-14

**Authors:** John Kim, Aaron S Kesselheim, Edward R Scheffer Cliff, Benjamin N Rome

**Affiliations:** Program On Regulation, Therapeutics, And Law (PORTAL), Division of Pharmacoepidemiology and Pharmacoeconomics, Department of Medicine, Brigham and Women’s Hospital and Harvard Medical School, Boston, MA 02120, UnitedStates; Program On Regulation, Therapeutics, And Law (PORTAL), Division of Pharmacoepidemiology and Pharmacoeconomics, Department of Medicine, Brigham and Women’s Hospital and Harvard Medical School, Boston, MA 02120, UnitedStates; Program On Regulation, Therapeutics, And Law (PORTAL), Division of Pharmacoepidemiology and Pharmacoeconomics, Department of Medicine, Brigham and Women’s Hospital and Harvard Medical School, Boston, MA 02120, UnitedStates; Program On Regulation, Therapeutics, And Law (PORTAL), Division of Pharmacoepidemiology and Pharmacoeconomics, Department of Medicine, Brigham and Women’s Hospital and Harvard Medical School, Boston, MA 02120, UnitedStates

**Keywords:** hyaluronidase, biologics, prescription drug policy, product hopping

## Abstract

Several intravenously administered biologic drugs have been reformulated with hyaluronidase to enable subcutaneous delivery. This offers greater convenience and fewer infusion reactions while also increasing overall spending if biosimilar competition for the subcutaneous versions begins later than for the original intravenous versions. As of December 2024, at least 9 biologics had investigational or approved hyaluronidase versions. Medicare spending on these drugs totaled $10.3B in 2022. For 4 of these drugs, hyaluronidase versions accounted for 5%-83% of Medicare spending in 2022, with hyaluronidase versions accounting for the highest share of spending for pertuzumab-trastuzumab and daratumumab and a lower share of spending for 2 drugs that had biosimilar competition for the original versions: rituximab and trastuzumab. The benefits of subcutaneous hyaluronidase versions must be balanced against the challenges that come with higher prices if these versions are introduced before biosimilar competition begins for the original versions. Policymakers should ensure manufacturers cannot use “hyaluronidase hopping” to delay biosimilar competition or eligibility for Medicare price negotiation.

In recent years, several intravenously administered biologic drugs have been reformulated with hyaluronidase. By breaking down local hyaluronans, hyaluronidase allows for subcutaneous uptake of large molecules that previously required intravenous infusions.^1^ Biologics reformulated with hyaluronidase can be more convenient for patients, associated with a faster administration times, and some are associated with a lower risk of infusion reactions compared to intravenous versions. The ease of administration for subcutaneous rather than intravenous versions could facilitate improved access and lower-cost delivery in community settings.

However, the introduction of hyaluronidase versions also leads to excess spending if sales of the branded product are maintained after the original intravenous versions face biosimilar competition. Manufacturers have previously used this strategy to successfully delay competition by introducing slightly modified versions of existing drugs.^2^ To understand the potential impact of “hyaluronidase hopping,” we investigated how hyaluronidase products have affected prescription drug spending in Medicare.

## Methods

In this cross-sectional study, we used databases of the pharmaceutical pipeline (Citeline Pharmaprojects) and US Food and Drug Administration (FDA) drug approvals (Drugs@FDA) to identify intravenously administered biologic drugs with approved or investigational hyaluronidase versions as of December 1, 2024. For investigational products, we used ClinicalTrials.gov to identify the stage of development (phase I/II/III trials). For FDA-approved versions, we calculated the number of years between the approval of the original version and the hyaluronidase version.

We used Medicare Part B and Part D public drug spending data to measure annual spending from 2014 to 2022 for the original drugs, hyaluronidase versions, and any FDA-approved biosimilar versions. We compared mean annual Medicare Part B spending per beneficiary for different versions of each drug. This study was not submitted for institutional review board review because it does not constitute human participants research under the Common Rule. Analyses were completed using Prism (GraphPad) and Excel (Microsoft).

## Results

We identified 9 biologics with hyaluronidase versions, including 7 that were approved by the FDA as of December 2024 and 2 that were in phase III trials (Table 1). The time between approval of the original and hyaluronidase versions ranged from 1 to 20 years. Medicare spending on these 9 drugs collectively was $10.3 billion in 2022, including $9.9 billion in Medicare Part B and $382 million in Medicare Part D.

**Table 1.** Intravenous biologics with approved or investigational hyaluronidase versions as of 2024

Original intravenous biologic (brand name)	Original FDA approval date	Selected indications	Medicare spending, 2022 ($, millions) ^a^	Name of hyaluronidase version	Hyaluronidase version status ^b^
Atezolizumab (Tecentriq)	May 2016	Multiple cancers	819	Tecentriq Hybreza	Approved Sep 2024
Daratumumab (Darzalex)	Nov 2015	Multiple myeloma	1990	Darzalex Faspro	Approved May 2020
Efgartigimod alfa (Vyvgart)	Dec 2021	Myasthenia gravis	154	Vyvgart Hytrulo	Approved Jun 2023
Nivolumab (Opdivo)	Dec 2014	Multiple cancers	1935	BMS-986298	Phase III, NCT05297565 ^c^
Ocrelizumab (Ocrevus)	Mar 2017	Multiple sclerosis	824	Ocrevus Zunovo	Approved Sep 2024
Pembrolizumab (Keytruda)	Sep 2014	Multiple cancers	5131	MK-3475A	Phase III, NCT04956692
Pertuzumab (Perjeta)	Jun 2012	Breast cancer	395	Pertuzumab/trastuzumab/ hyaluronidase-zzxf (Phesgo) ^d^	Approved Jun 2020
Rituximab (Rituxan)	Nov 1997	Non-Hodgkin lymphoma, autoimmune conditions	1169	Rituxan Hycela	Approved Jun 2017
Trastuzumab (Herceptin)	Sep 1998	Breast cancer, gastric cancer	420	Herceptin Hylecta	Approved Feb 2019

^a^Includes Medicare Part B spending plus estimated net Medicare Part D spending after subtracting 4-quarter rolling average non-Medicaid rebate estimates from SSR Health. Includes spending on the original version, hyaluronidase versions, and any FDA-approved biosimilars.

^b^As of December 1, 2024, we queried the Citeline Pharmaprojects database with the search words “hyaluronidase” or “subcutaneous.” We identified valid search hits, which represented trials of existing, FDA-approved biologics co-formulated with hyaluronidase products. We verified the status of all clinical trials using clinicaltrials.gov.

^c^This phase III trial was completed on February 8, 2024.

^d^According to the FDA labeling, pertuzumab should only be used in combination with trastuzumab; the hyaluronidase version combines both drugs into a single fixed-dose combination.

**Figure 1.**
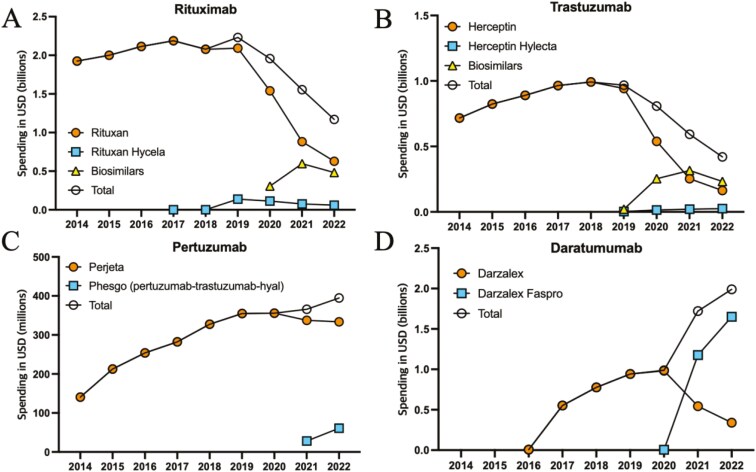
Medicare spending on 4 biologics with hyaluronidase versions, 2014-2022.* * Includes Medicare Part B spending plus estimated net Medicare Part D spending on rituximab (A), trastuzumab (B), pertuzumab (C), and daratumumab (D) after subtracting 4-quarter rolling average non-Medicaid rebate estimates from SSR Health. Spending was converted to 2022 US dollars using the Consumer Price Index for All Urban Consumers. Spending on the combined pertuzumab-trastuzumab hyaluronidase version did not account for potential trastuzumab savings, because patients using the subcutaneous combined formulation would have otherwise received intravenous pertuzumab and trastuzumab separately.

Rituximab and trastuzumab had biosimilar intravenous versions launched within 3 years after the hyaluronidase version; in these cases, the hyaluronidase versions accounted for 5.3% of spending in 2022 (Figure 1A and B). From 2019 to 2022, Medicare spending on all rituximab products decreased by 47.6%, from $2.2 billion to $1.2 billion. Spending on trastuzumab products, excluding products combined with pertuzumab, declined by 56.6% from 2019 to 2022, from $967 million to $420 million. In both cases, these spending decreases corresponded with emerging biosimilar competition.

By contrast, hyaluronidase versions accounted for 15.5% of Medicare pertuzumab spending and 82.9% of daratumumab spending (Figure 1C and D). These versions were both approved in 2020 and neither of the original drugs had approved biosimilar versions by December 2024. From 2020 to 2022, annual spending on pertuzumab-containing products increased by 10.9%, from $356 million to $395 million, and daratumumab-containing product spending increased by 101.5%, from $988 million to $2.0 billion.

For rituximab and trastuzumab, the mean annual Medicare Part B spending per beneficiary in 2022 was lower for the hyaluronidase versions than the original biologic; biosimilar versions for both drugs had even lower spending. Annual spending per beneficiary was $21 071 for brand-name intravenous rituximab, $19 762 for the hyaluronidase version, and ranged from $12 913 to $14 410 for biosimilars. The annual cost for hyaluronidase versions was higher than the original versions for pertuzumab ($52 574 vs $45 864) and daratumumab ($87 693 vs $64 164).

## Discussion

Seven intravenous biologic medications have subcutaneous formulations with hyaluronidase available for US patients, with several more likely to become available soon. In 2 cases, hyaluronidase versions accounted for a sizeable share of spending on these products in 2022, suggesting that patients may be shifting toward using the subcutaneous versions in the absence of biosimilar competition for those drugs. Hyaluronidase versions are generally approved several years after the original drug, raising concerns that “hyaluronidase hopping” could be used by manufacturers to delay eventual biosimilar competition, and thus lead to excess spending by patients and the healthcare system.

Hyaluronidase versions may offer clinical benefits to patients. For example, compared to intravenous daratumumab, subcutaneous daratumumab-hyaluronidase was associated with lower rates of infusion reactions (34.5% vs 12.7%) and serious infusion reactions (5.4% vs 1.5%); to reduce the risk of such reactions, intravenous daratumumab was diluted and administered slowly, which made administration inconvenient for patients and infusion centers.^3^ By contrast, the subcutaneous and intravenous versions of rituximab had similar rates of adverse events, and injection-related reactions were more common for those receiving the subcutaneous version.^4^

Hyaluronidase hopping could lead to excess healthcare spending by undermining biosimilar competition for the original intravenous biologic versions, because hyaluronidase-enhanced versions may be protected by newer patents and can earn 12-year market exclusivity periods free from biosimilar competition under the Biologics Price Competition and Innovation Act of 2010. In some cases, we found that newer hyaluronidase versions had similar or lower costs than the intravenous version, but even in these cases, the newer version could lead to higher spending if patients use the brand-name hyaluronidase version even after less costly biosimilars of the original become available.

Other approaches through which drug companies have launched newer formulations of existing brand-name drugs have led to billions of dollars in excess spending.^2,5^ A notable example was ocrelizumab, an anti-CD20 monoclonal antibody that was 4-8 times costlier than rituximab and introduced by the same manufacturer shortly before rituximab faced biosimilar competition.^6^ From our data, it appears that the introduction of a hyaluronidase version of a biologic is most likely to have a major market impact when it is introduced more than 3 years before the introduction of biosimilar versions of the original product or when it has meaningful clinical benefits compared to the original version, as was the case for daratumumab. This may be a leading reason why the manufacturer of pembrolizumab, the world’s top-selling drug in 2024, plans to launch a subcutaneous hyaluronidase version in the United States as soon as October 2025, which could extend sales of the blockbuster drug even after key patents expire in 2028.^7^

Another concern is that hyaluronidase hopping could delay savings from Medicare negotiation. Under the Inflation Reduction Act, Medicare will soon begin negotiating lower prices for eligible biologic drugs reimbursed under Part B; biologic drugs become eligible for negotiation 11 years after FDA approval. Under current guidance, the eligibility of products that combine multiple active ingredients will be assessed separately from products that contain each ingredient separately, which means that price negotiation for hyaluronidase versions might be delayed for years after the negotiation of intravenous versions.^8^ In  Draft Guidance published in May 2025, however, the Centers for Medicare and Medicaid Services solicited comments about how to could combine products containing multiple ingredients, not all of which are therapeutically active.^9^  Such a change is important for allowing later-introduced hyaluronidase formulations to qualify for negotiation coincident with the original version.

This study has several limitations. As Part D rebates are generally confidential, we estimated net spending, although most cancer drugs have small rebates.^10^ We focused on Medicare spending; although this represents a large share of United States spending for cancer drugs, we did not account for additional spending for those with commercial insurance or other public insurance programs like Medicaid. Our analysis did not account for potential savings associated with reduced time and cost for administering subcutaneous injections rather than intravenous infusions. This transition could lead to direct healthcare savings due to lower administration fees and indirect savings from less patient and caregiver time spent receiving infusions.

Reformulating biologics with hyaluronidase for subcutaneous delivery provides convenience and, in some cases, clinical benefits. But policymakers should ensure that “hyaluronidase hopping” does not delay cost-saving measures like biosimilar competition or Medicare price negotiation.

## Data Availability

All data are publicly available.
